# Double Trouble: A Case Report of Bilateral Tubal Ectopic Pregnancy After Intrauterine Insemination Along With a Literature Review

**DOI:** 10.7759/cureus.79294

**Published:** 2025-02-19

**Authors:** Rodrigo Realista, Ana Calhau, Isabel Pereira

**Affiliations:** 1 Obstetrics and Gynaecology, Centro Hospitalar São João, Porto, PRT; 2 Obstetrics and Gynaecology, SESARAM (Serviço de Saúde da Região Autónoma da Madeira, EPERAM) - Hospital Dr. Nélio Mendonça, Funchal, PRT

**Keywords:** bilateral tubal ectopic pregnancy, controlled ovarian stimulation, intrauterine insemination, pelvic ultrasound, ruptured tubal ectopic pregnancy

## Abstract

Bilateral tubal ectopic pregnancy (BTP) is a rare variant of ectopic pregnancy (EP). Besides sharing the same risk factors, the similarity in clinical presentation between BTP and EP hamper the diagnosis, often made only during surgery. The management of BTP is complex, raising considerations for fertility-preserving surgery. While evidence is limited, salpingostomy is typically preferred over salpingectomy, as it does not compromise fertility outcomes.

The document includes a case report of a 40-year-old woman with a surgically diagnosed and treated BTP five weeks after an intrauterine insemination procedure with ovarian stimulation. Diagnostic laparoscopy revealed a ruptured right fallopian tube and a mass in the left fallopian tube, despite no prior indications of left-side abnormalities. A right salpingectomy was performed. Post-surgery, a follow-up ultrasound showed a left para-adnexal mass, and beta-human chorionic gonadotropin (beta-HCG) levels fluctuated, initially decreasing but later rising, prompting the need for surgical treatment.

This case report emphasizes the need for careful management and thorough inspection of both adnexal areas to improve recognition and care.

## Introduction

An ectopic pregnancy (EP) is defined by the implantation of the gestational sac in any site other than the uterus; it occurs in 1.5-2% of all pregnancies [1]. A bilateral tubal ectopic pregnancy (BTP) is the rarest form of ectopic pregnancy, with an incidence of 1:200 000 pregnancies, and is characterized by the implantation of one gestational sac in each fallopian tube (1). Despite the rarity of BTP, it shares the same risk factors as EP: sexually transmitted diseases; pelvic inflammatory infections; history of previous EPs; tubal surgery; infertility (probably reflecting the higher incidence of tubal abnormalities in this subgroup), and clomiphene citrate stimulation [2-4].

Despite the uncertainty around BTP etiology, it is hypothesized that it can have its origin in the transperitoneal migration of trophoblastic cells [5]. Other theories have been postulated, suggesting the possibility of multiple ovulations and implantation on damaged sites [6] or even superfetation (implantation of an ovum in an already pregnant woman) [5].

Despite the similarity in clinical presentation with EP, BTP is usually challenging to distinguish unless a careful examination of both adnexal areas is performed, and sometimes, it is only suspected during laparoscopic exploration [5]. Because of bilateral tubal involvement, surgical management of these situations requires special considerations, especially, if possible, fertility-preserving surgery. There is little evidence to support salpingostomy over salpingectomy [7]. Nevertheless, salpingostomy is usually preferred since it allows the preservation of both fallopian tubes, allowing better fertility prospects. 

We present a case report of a BTP, surgically diagnosed and managed, and intend to add more information to the sparse literature, facilitating decision-making in the surgical management of these cases.

## Case presentation

A 40-year-old patient with a history of unexplained primary infertility presented at our emergency ward with light vaginal bleeding. The patient’s past health history had no event to notice, and she had no history of gynecological surgery, sexually transmitted disease, or pelvic inflammatory disease. She had previously been submitted to two unsuccessful intrauterine inseminations (IUIs), both with ovarian stimulation with recombinant follicle-stimulating hormone (FSH) (75 IU/day).

**Figure 1:**
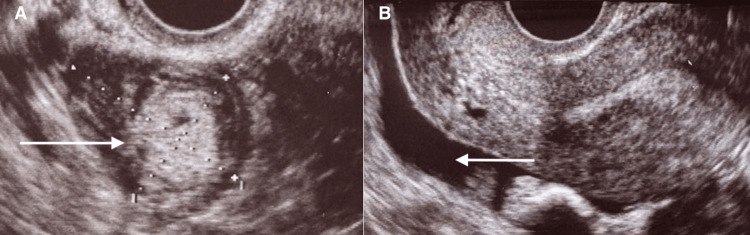
Ultrasound view before surgical exploration 1A: a right-side adnexal mass suggestive of ectopic pregnancy; 1B: sagittal view showing fluid accumulation in the pelvic cavity

One month before admission, the patient underwent one cycle of ovarian-controlled stimulation with recombinant FSH (75IU/day), starting on day 2 of the menstrual cycle. She had two main follicles: 15 mm on the left ovary and 19 mm on the right ovary, and was triggered with recombinant alfa chorionic gonadotrophin (250μg) to be submitted to an IUI. She missed the beta-human chorionic gonadotropin (beta-HCG) control testing due to personal issues and was admitted to the emergency department five weeks after the IUI with vaginal bleeding. Apart from the vaginal bleeding, she had no other complaints and was conscious and oriented. Abdominal examination was normal, with no pain or tenderness. Vaginal examination presented no fornicial tenderness or palpable mass. Her blood pressure was normal, as well as her heart rate. On physical examination, she had generalized discomfort in her abdomen, with no tenderness. Transvaginal ultrasound of the pelvis on admission showed an 11-mm endometrium with no intracavitary gestational sac and a hyperechogenic right-sided para-adnexal neoformation measuring 36 mm x 28 mm (Figure 1A), with a moderate amount of free fluid in the pelvic cavity (Figure 1B). There was no mention of left-side findings.

Blood tests showed a beta-HCG level of 11 173 mIU/mL (< 2 mIU/mL) and a hemoglobin level of 12,1 g/dL, with a further decrease to 11.1 g/dL (12.1-15.1 g/dL) a few hours later, with no objective hemodynamic instability (blood pressure of 113/78 mmHg and heart rate of 79 bpm).

**Figure 2:**
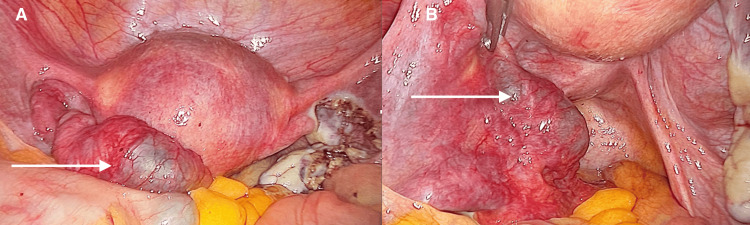
Laparoscopic image of the right adnexal area In panels 2A and 2B, there is evidence of an enlarged left fallopian tube after the salpingectomy of the ruptured right fallopian tube.

Diagnostic laparoscopy was offered to the patient in view of the suspicion of right tubal ectopic pregnancy. Laparoscopy showed a small quantity of hemoperitoneum of 100 mL, a normal uterus, and a right ruptured fallopian tube with an ectopic gestational sac in active bleeding. In the left adnexal area, an image of a dilated, enlarged, and hypervascularized left fallopian tube (Figures 2A, 2B).

Since there was no previous mention of abnormal ultrasound findings of the left adnexal area, and according to the previously signed informed consent in which the patient only allowed the removal of the right fallopian tube, right salpingectomy was performed, with the extraction of the fallopian tube inside an endobag. The patient made an uneventful postoperative recovery and her hemoglobin was stable. 

**Figure 3:**
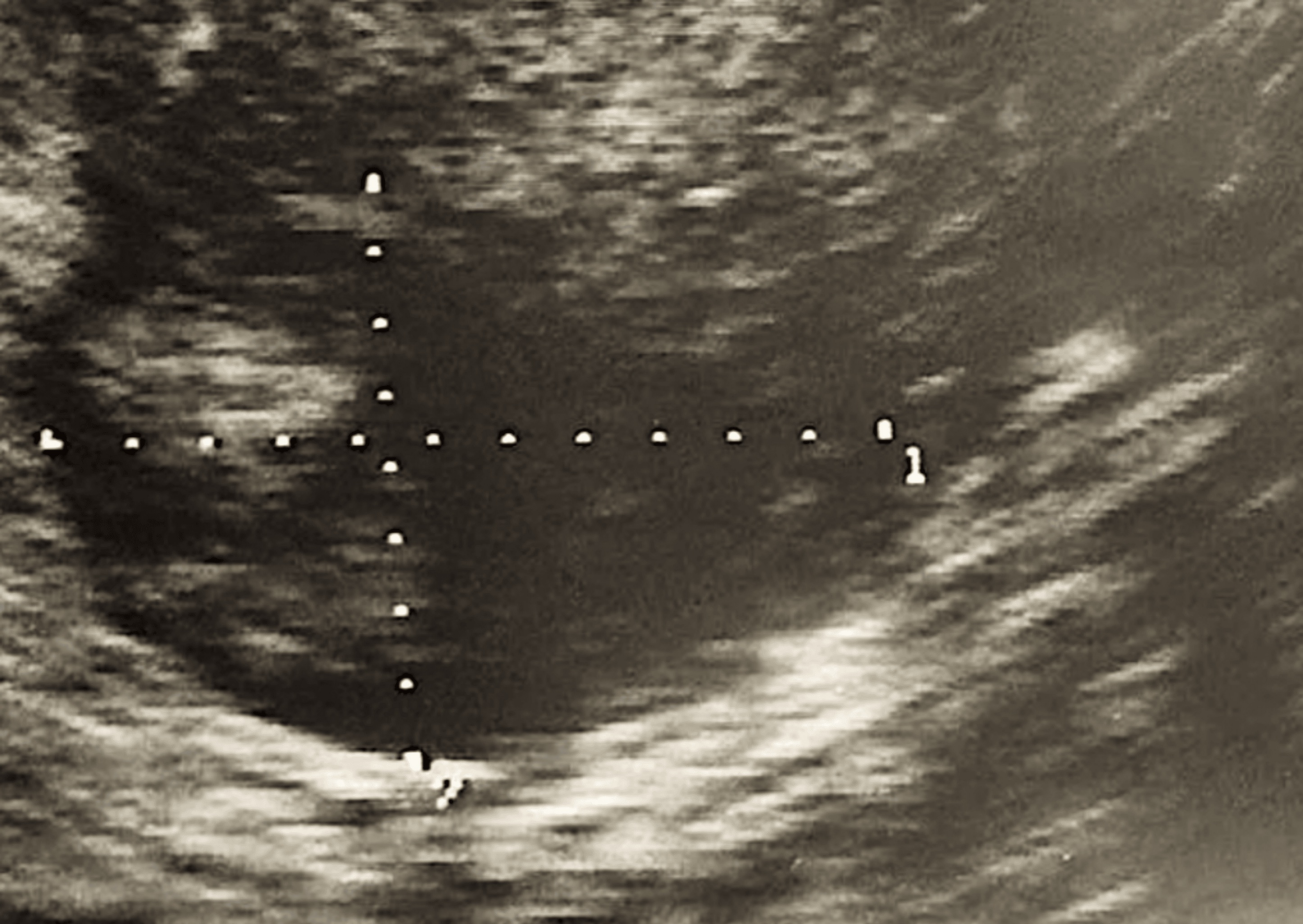
Ultrasound view of the left adnexal area based on the suspicion raised during the first surgery

Based on the laparoscopic findings, a postoperative ultrasound was performed, in which a left para-adnexal heterogenous mass measuring 27 x 20 mm was present (Figure 3).

Beta-HCG on day 2 after surgery showed a decrease from the previous 11173 mIU/mL to 9578 mIU/mL. The patient was stable, with no complaints and minimal vaginal bleeding. On day 3 after surgery, beta-HCG began to rise to a maximum of 13000 mIU/mL at day 4. 

**Figure 4:**
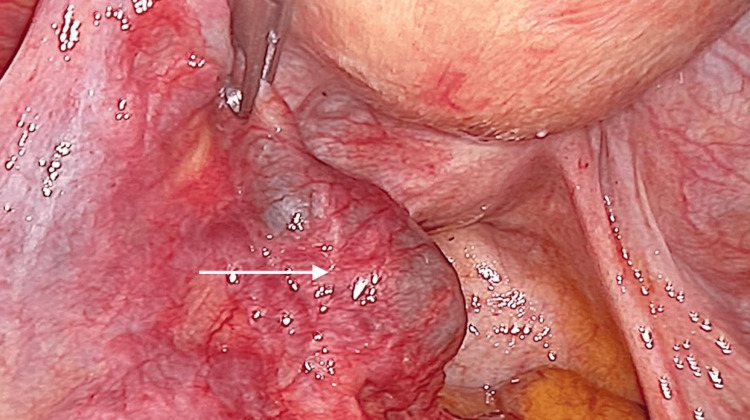
Left fallopian tube during the second surgery

A new diagnostic laparoscopy was performed, this time with no hemoperitoneum seen but with a larger left fallopian tube (Figure 4).

A left salpingectomy was performed. The patient was discharged on day 2 after the second laparoscopy. Histologic findings confirmed the diagnosis of BTP.

## Discussion

EPs are one of the most serious conditions in obstetrics and frequently constitute obstetric emergencies due to their potentially life-threatening consequences. Despite the rareness of BTP, a rise in its occurrence has been observed in recent years, probably associated with an increase in assisted reproductive techniques (ART). Concerning our patient, the fertility treatment with controlled ovarian stimulation and the presence of two dominant follicles probably increased the risk of EP.

The risk of ectopic pregnancy associated with ART has been discussed by many authors. Clayton et al. claimed that ART procedures were an overall risk factor for EP, and they suggested that ART patients with tubal factor infertility were more predisposed to EP due to their impaired tubal function [8]. However, patients with non-tubal female factors of infertility also conceived more ectopically (odds ratio (OR) 1.4, 95% confidence interval (CI) 1.2-1.6). Most of the studies share the same downfall, which is the inability to rule out the impact of the infertile background on women who had ART [9]. Therefore, some researchers doubted the conclusion that ART promoted ectopic pregnancies [10].

Protocols with clomiphene citrate have been shown to independently increase the risk of EP, but there is evidence demonstrating that protocols with gonadotrophin-releasing hormone analogs (GnRHa) did not show any increased risk [3]. 

In the presented case, the ovarian stimulation before IUI was successful and led to the development of two main follicles, one in each ovary. Due to the limited efficacy of IUI in achieving a term pregnancy, and the fact that she had two previous unsuccessful IUIs, it was decided to proceed with the technique.

BTP is clinically indistinguishable from EP and patients present with the classic triad of pain, vaginal bleeding, and amenorrhea [11]. Although crucial in the diagnosis and management of EP, beta-HCG serum levels have no utility in the differential diagnosis between EP and BTP.

Most cases of EP are diagnosed based on the absence of intrauterine pregnancy and a positive beta-HCG serum level, rather than the ultrasound visualization of a gestational sac, and the fact that the clinical presentation of BTP mirrors that of unilateral EP raises the importance of careful ultrasound examination to assess both fallopian tubes [12]. The lack of awareness of BTP, in part due to its rarity or sometimes the obscured visibility from the presence of blood in the pelvis, further hampers the ultrasound diagnosis. In fact, one literature review concluded that only around 15% of BTP were diagnosed previously on ultrasound [13]. Therefore, the diagnosis is typically made during diagnostic laparoscopy.

The management of BTP requires careful consideration since both fallopian tubes are involved with possible negative consequences on future fertility outcomes. There are no guidelines available for the management of BTP. In the presented case, surgical management was decided since the elevated beta-hCG level (above 5000 mIU/mL) and the decrease in hemoglobin level shown in a couple of hours (around 1 g/dL) are relative contraindications to medical therapy.

The decision to perform salpingostomy versus salpingectomy varies depending on the extent of the fallopian tube damage at the time of diagnosis and the patient’s desire for future fertility [14]. Salpingostomy consists of the section of the affected fallopian tube portion and aspiration of the content inside the tube, allowing tubal preservation, while salpingectomy consists of the removal of the affected fallopian tube.

Although there is little evidence to support salpingostomy over salpingectomy, the first one is usually preferred since it is assumed that the preservation of both tubes offers better fertility prospects [1].

One study reviewing the pregnancy outcomes after surgical management of EP found that in the patient subgroup with a prior history of fertility-reducing factors, salpingostomy was associated with better rates of future intrauterine pregnancy [15]. 

A randomized controlled trial including 446 patients, with 215 patients allocated to Salpingostomy and 231 to salpingectomy, concluded that salpingostomy does not significantly improve fertility prospects compared with salpingectomy [7]. They also found comparable rates of repeat ectopic tubal pregnancies in both groups.

In our case, the decision to proceed with salpingectomy during the first surgery was straightforward because the right fallopian tube was ruptured. Since there was no previously signed informed consent regarding intervention in the left fallopian tube and it was not ruptured, we decided to proceed with conservative management to make sure it was a BTP. Our decision was also based on the patient’s age since in Portugal, assisted reproduction techniques other than embryo transfer are only allowed until the age of 40 in the public system, which led us to be extremely cautious with fertility preservation.

The beta-HCG rise after surgery corroborated the presence of an EP on the left tube, and management was carefully discussed with the couple. We decided to proceed with left salpingectomy in view of a future in vitro fertilization/intra-cytoplasmatic sperm injection technique to raise the chance of a successful pregnancy.

Our case underlines the importance of a careful ultrasound examination of both adnexal areas when a suspicion of EP is raised. It is important to be aware of this rare entity since surgical management can compromise fertility prospects.

The appropriate surgical procedure after an incidental finding of unilateral or bilateral ectopic pregnancy must be considered in these patients, and the choice of fertility-preserving surgery must be weighed against the increased risk of recurrence. 

The limitation of our patient management might have been related to the model of the ultrasound device, and it seems that the quality and model of the ultrasound device have a significant impact on diagnosis.

Due to the absence of available algorithms for BTP treatment, a model has been proposed by Benz et al. in 2022, in which the management options available are decided first considering the hemodynamic stability of the patient, and in stable patients, a decision should be made considering the desire for future fertility [12].

A literature review was performed, in which we included all the published BTP case reports in the last two years. The results are presented in Table 1. We found five published cases of BTP, in which most of the BTP cases were misdiagnosed during an ultrasound examination. Trefsgar JR et al. reported BTPs during laparoscopic exploration for suspected unilateral ectopic pregnancy [16]. Mansouri Z et al. described a case in which a diagnosis was made during postoperative follow-up [17]. Demircioglu F et al. also reported BTPs during laparoscopic exploration for suspected unilateral EP [18]. In a case published by Farshidpour LS et al., the patient was discharged to enter an emergency setting in a different clinic with a contralateral EP 11 days after the first surgery [19]. There was only one case in which a diagnosis was made based on ultrasound and before surgery [20]. Most of the cases were managed surgically, with salpingectomy or salpingostomy being performed according to the fertility desire of the patient or the extent of tubal damage.

**Table 1:** Reported cases of bilateral tubal pregnancy published in the past two years STDs: sexually transmitted diseases; MTX: methotrexate

Author	Year	EP Risk Factors	Diagnosis	Treatment
Trefsgar, JR., et al. [16]	2024	No risk factors	Diagnostic laparoscopy	Bilateral salpingectomy
Mansouri, Z., et al. [17]	2023	No risk factors	Postoperative follow-up	Lapasocopic salpingostomy + MTX
Demircioglu, F. et al. [18]	2023	No risk factors	Diagnostic laparoscopy	Bilateral laparoscopic salpingectomy
Farshidpour, LS., et al. [19]	2023	Personal history of STDs	Postoperative follow-up	Bilateral salpingectomy
Eghbali, E., et al. [20]	2023	No risk factors	Ultrasound	Bilateral laparoscopic salpingostomy

All of the presented case reports highlighted the crucial role of a high suspicion index and a careful ultrasound examination of both fallopian tubes. Further studies are needed to best evaluate the outcomes in patients with BTP.

## Conclusions

BTP represents a clinical challenge as the presentation is quite similar to that of unilateral ectopic pregnancy and is easily missed. Furthermore, there are no treatment guidelines or protocols available for its management. Our case report adds to the literature by raising awareness of this rare entity and showing that careful management and detailed inspection of both adnexal areas are required in these cases.
